# Identification of Neural Crest and Glial Enhancers at the Mouse *Sox10* Locus through Transgenesis in Zebrafish

**DOI:** 10.1371/journal.pgen.1000174

**Published:** 2008-09-05

**Authors:** Anthony Antonellis, Jimmy L. Huynh, Shih-Queen Lee-Lin, Ryan M. Vinton, Gabriel Renaud, Stacie K. Loftus, Gene Elliot, Tyra G. Wolfsberg, Eric D. Green, Andrew S. McCallion, William J. Pavan

**Affiliations:** 1 Genome Technology Branch, National Human Genome Research Institute, National Institutes of Health, Bethesda, Maryland, United States of America; 2 McKusick–Nathans Institute of Genetic Medicine, Department of Molecular and Comparative Pathobiology, The Johns Hopkins University School of Medicine, Baltimore, Maryland, United States of America; 3 Genetic Disease Research Branch, National Human Genome Research Institute, National Institutes of Health, Bethesda, Maryland, United States of America; Stanford University School of Medicine, United States of America

## Abstract

*Sox10* is a dynamically regulated transcription factor gene that is essential for the development of neural crest–derived and oligodendroglial populations. Developmental genes often require multiple regulatory sequences that integrate discrete and overlapping functions to coordinate their expression. To identify *Sox10* cis-regulatory elements, we integrated multiple model systems, including cell-based screens and transposon-mediated transgensis in zebrafish, to scrutinize mammalian conserved, noncoding genomic segments at the mouse *Sox10* locus. We demonstrate that eight of 11 *Sox10* genomic elements direct reporter gene expression in transgenic zebrafish similar to patterns observed in transgenic mice, despite an absence of observable sequence conservation between mice and zebrafish. Multiple segments direct expression in overlapping populations of neural crest derivatives and glial cells, ranging from pan-*Sox10* and pan-neural crest regulatory control to the modulation of expression in subpopulations of *Sox10-*expressing cells, including developing melanocytes and Schwann cells. Several sequences demonstrate overlapping spatial control, yet direct expression in incompletely overlapping developmental intervals. We were able to partially explain neural crest expression patterns by the presence of head to head SoxE family binding sites within two of the elements. Moreover, we were able to use this transcription factor binding site signature to identify the corresponding zebrafish enhancers in the absence of overall sequence homology. We demonstrate the utility of zebrafish transgenesis as a high-fidelity surrogate in the dissection of mammalian gene regulation, especially those with dynamically controlled developmental expression.

## Author Summary

The neural crest is a population of embryonic migratory stem cells. They form atop the future spinal cord and migrate throughout developing embryos and form many different cells, including the epidermal pigment cells, bone cells in the head, and nerve cells of the peripheral nervous system. In this study, we studied the genome elements responsible for expression of *SOX10*, a dynamically expressed gene that is essential for neural crest development. We isolated candidate regulatory elements for *SOX10* by identifying the small percentage of genomic DNA around the gene that did not vary as avian and mammalian genomes changed though evolution. We tested these fragments for their ability to regulate gene expression in zebrafish, a model system that is highly efficient for DNA-mediated expression studies and embryology. We found that even though the genome sequences were not similar to the *SOX10* gene in fish, the genomic fragments were able to recapitulate the dynamic expression of *SOX10* during development. Through computational analysis of the sequences, we identified a transcription factor binding site signature that identified the corresponding zebrafish *SOX10* regulatory elements. This study describes a paradigm for dissecting regulation of essential genes that display complex expression patterns during development.

## Introduction

The neural crest is a transient migratory population of cells that arise at the dorsal aspect of the neural tube as it closes and that give rise to myriad structures including, but not limited to, melanocytes, enteric nervous system (ENS), and myelinating Schwann cells [1]. *SOX10* (SRY-box containing gene 10) encodes a critical transcription factor in neural crest development [2],[3]. All neural crest cells express SOX10 upon emigration throughout the early stages of their respective journeys. Subsequently, expression is down-regulated in all neural crest–derived cells with population-specific timing. SOX10 expression is maintained in melanocytes (mouse) and Schwann cells (mouse and zebrafish) [2],[3],[4],[5],[6],[7],[8]. Additionally, SOX10 is expressed in presumptive oligodendrocytes; signal increases in concert with the onset of myelination and is maintained thereafter [2],[6],[9],[10]. Naturally occurring and induced mutations in animal models and spontaneous mutations in the human population exemplify the developmental requirement for *Sox10* during development. Human loss-of-function *SOX10* mutations result in Waardenburg-Shah Syndrome (WS4; OMIM Accession No. 277580), a disorder characterized by impaired pigmentation and aganglionic megacolon consistent with Hirschsprung's Disease [11],[12],[13],[14]. Furthermore, dominant-negative *SOX10* mutations have been identified in patients with PCWH (OMIM Accession No. 609136), a complex syndrome characterized by the co-presentation of a WS4-like phenotype with central dysmyelinating leukodystrophy and demyelinating peripheral neuropathy consistent with Charcot-Marie-Tooth disease type I [11],[15].

Similarly, studies in zebrafish and mice demonstrate the broad need for SOX10 during vertebrate development [4],[8],[16],[17]. A missense mutation in the zebrafish *sox10* coding sequence results in dramatic hypopigmentation accompanied by a reduction in enteric nervous system neuronal and Schwann cell populations [16],[17]. In a similar fashion, mutations in the mouse *Sox10* gene yield mice with impaired pigmentation and megacolon, and such models have been critical for understanding the expression and function of *Sox10* [4],[18]. Indeed, in a recent report we identified a deletion of approximately 16 kb residing approximately 47 kb upstream of *Sox10*, resulting in the *Sox10^Hry^* mouse model of WS4 [19]. Although these mice present with enteric and pigmentary deficits, this deletion severely disrupts, but does not ablate, developmental expression of *Sox10*. These data suggested that the deletion harbors a subset of cis-acting transcriptional regulatory elements necessary for *Sox10* expression, and our functional analyses revealed at least one highly conserved segment within the deleted region that directs reporter gene expression in cultured melanocytes [19]. Comprehensive dissection of the cis-acting transcriptional regulatory sequences at *SOX10* will be important for understanding the dynamic control of this transcription factor in specific neural crest derivatives and in oligodendrocytes, as well as for assessing the role of non-coding mutations in disease susceptibility in which *SOX10* may play a role. Furthermore, these efforts will provide a significant first step in establishing the sequence substrates necessary for the development of cell-specific regulatory vocabularies.

Here, we report a comprehensive analysis of highly conserved, non-coding sequences at the mouse *Sox10* locus, integrating *in vitro* cell studies in multiple cell lines and transgenesis in developing zebrafish and mice. Our results indicate that regulatory control at this locus coordinates information from a wide array of sequences whose independent regulatory contributions range from discrete sub-populations of crest derivatives to near pan-neural crest or pan-Sox10 expression. Critically, our data highlight the power of our zebrafish transgenic assay, demonstrating that it provides a high fidelity read out for the regulatory function of conserved non-coding *Sox10* mouse sequences even in the absence of overt sequence conservation. Our results are largely consistent with recent preliminary analysis of a subset of *Sox10* sequences in mouse, including the identification of regulatory elements that direct expression in peripheral neuronal populations and non-ectomesenchymal crest derivatives. Where differences are evident, they are largely accounted for by incomplete overlap among assayed sequences or the ability of dynamic analyses in zebrafish to uncover reporter expression that is not evident in Sox10 positive populations during mammalian development. Consequently, we also provide the first report of sequences directing expression in developing melanocytes and myelinating glia, for which no previous report exists. Additionally, our data also revealed regulatory segments that function in the same cell types but within different developmental time intervals. Finally, we demonstrate that two identified regulatory sequences with pan-neural crest regulatory control harbor dimeric SoxE binding sites that account for a significant fraction of their regulatory integrity, and use this data to identify two regulatory sequences at the zebrafish *sox10* locus that we propose to be their functional orthologs.

## Results

### Conserved, Non-Coding Sequences at *Sox10* Display Enhancer Activity in Neural Crest–Derived Cells

**Figure 1:**
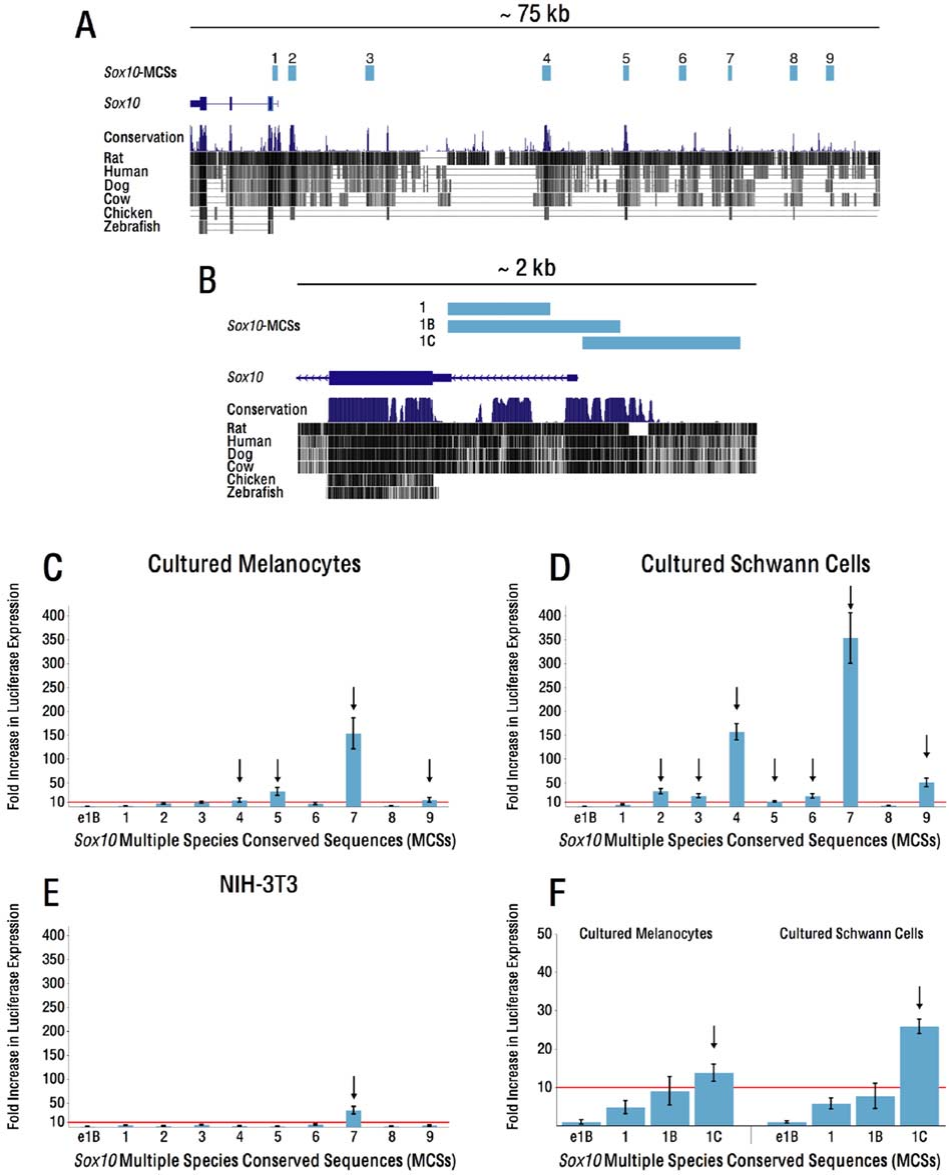
*Sox10*-MCSs display enhancer activity in Neural Crest–derived cell lines. A) *Sox10*-MCSs (pale blue bars) identified within and upstream of the *Sox10* locus, depicted by a 75 kb interval from the UCSC Genome Browser (genome.ucsc.edu). B) Enlargement of a 2 kb interval encompassing the *Sox10* transcriptional start site. Three elements, comprising sequence from within intron one alone (*Sox10*-MCS1), intron one and sequence 5′ of the TSS (*Sox10*-MCS1B), and sequence only 5′ of the TSS (*Sox10*-MCS1C) were subcloned and tested individually for enhancer activity. C–F) *Sox10*-MCSs cloned upstream of a minimal promoter driving luciferase reporter expression were assayed for enhancer activity in melan-a cells (C and F), S16 cells (D and F), and NIH-3T3 cells (E). The red line demarcates the 10-fold cut-off for “enhancer activity” (see Results); arrows indicate sequences exceeding that activity threshold. Error bars, SD (standard deviation).

Evolutionary sequence conservation is a robust metric for the identification of putatively functional sequences [20],[21],[22]. In a recent study, we identified nine highly-conserved genomic segments 5′ to the mouse *Sox10* coding exons [19]. We termed these *Sox10*-MCSs (for Multiple-species Conserved Sequences; Figure 1A and Table 1). To determine their regulatory potential, we have assayed each segment *in vitro*, and *in vivo* through transgenesis in vertebrate systems. In addition, to more closely examine the genomic region surrounding the *Sox10* transcriptional start site (TSS), we also assayed a genomic segment that harbors a conserved intron one element (*Sox10*-MCS1, Figure 1B), another that extends 5′ to the TSS (*Sox10*-MCS1B, Figure 1B), and another harboring only sequences upstream of the TSS (*Sox10*-MCS1C, Figure 1B).

**Table 1:** Genomic segments analyzed in this study.

Genomic Segment	Coordinates at UCSC Genome Browser^1^
Sox10-MCS1	chr15:78,991,037–78,991,472
Sox10-MCS1B	chr15:78,991,037–78,991,759
Sox10-MCS1C	chr15:78,991,602–78,992,260
Sox10-MCS2	chr15:78,992,786–78,993,476
Sox10-MCS3	chr15:79,001,113–79,001,872
Sox10-MCS4	chr15:79,020,080–79,020,899
Sox10-MCS5	chr15:79,028,761–79,029,307
Sox10-MCS6	chr15:79,034,832–79,035,411
Sox10-MCS7	chr15:79,040,107–79,040,404
Sox10-MCS8	chr15:79,046,712–79,047,379
Sox10-MCS9	chr15:79,050,690–79,051,355
zf-sox10-E1	chr13:15,933,435–15,933,953
zf-sox10-E2	chr13:15,949,868–15,950,386
zf-sox10-E3	chr13:15,992,412–15,992,923

1 Coordinates are from the February 2006 UCSC Genome Browser Mouse assembly and the March 2006 UCSC Genome Browser Zebrafish assembly for mouse and zebrafish genomic sequences, respectively.

We first tested the ability of each *Sox10*-MCS to direct reporter gene expression in two relevant cell lines. Briefly, we cloned each mouse genomic segment (Figure 1A and 1B) upstream of a minimal promoter (pe1B) directing basal luciferase expression and transfected them independently into two cell lines that express *Sox10* [cultured melanocytes (melan-a cells) and cultured Schwann cells (S16 cells)], and one in which *Sox10* is not expressed (NIH-3T3 cells). To enrich for authentic enhancers, we defined “enhancer activity” as segments that enhance reporter gene expression at a level 10-fold or higher compared to the control (pe1B in Figure 1C through E). One segment (*Sox10*-MCS7) showed enhancer activity in all three cell lines (Figure 1C through E), three segments (*Sox10*-MCS4, *Sox10*-MCS5, and *Sox10*-MCS9) showed enhancer activity only in the neural crest–derived cultured melanocytes and Schwann cells (Figure 1C and D), and three segments (*Sox10*-MCS2, *Sox10*-MCS3, and *Sox10*-MCS6) showed enhancer activity only in cultured Schwann cells (Figure 1D). Interestingly, while neither *Sox10*-MCS1 nor *Sox10*-MCS1B displayed enhancer activity, *Sox10*-MCS1C directed reporter gene expression in both melanocytes and Schwann cells (Figure 1F). These data suggest that *Sox10*-MCS1C may represent a proximal *Sox10* enhancer element. In total, 8 of the 11 genomic fragments studied displayed enhancer activity in *Sox10* expressing cell lines, suggesting that the *Sox10* locus does harbor multiple, coordinated cis-acting regulatory elements.

### *Sox10*-MCSs Direct Tissue-Specific Reporter Gene Expression in Transgenic Zebrafish Embryos

**Figure 2 pgen-1000174-g002:**
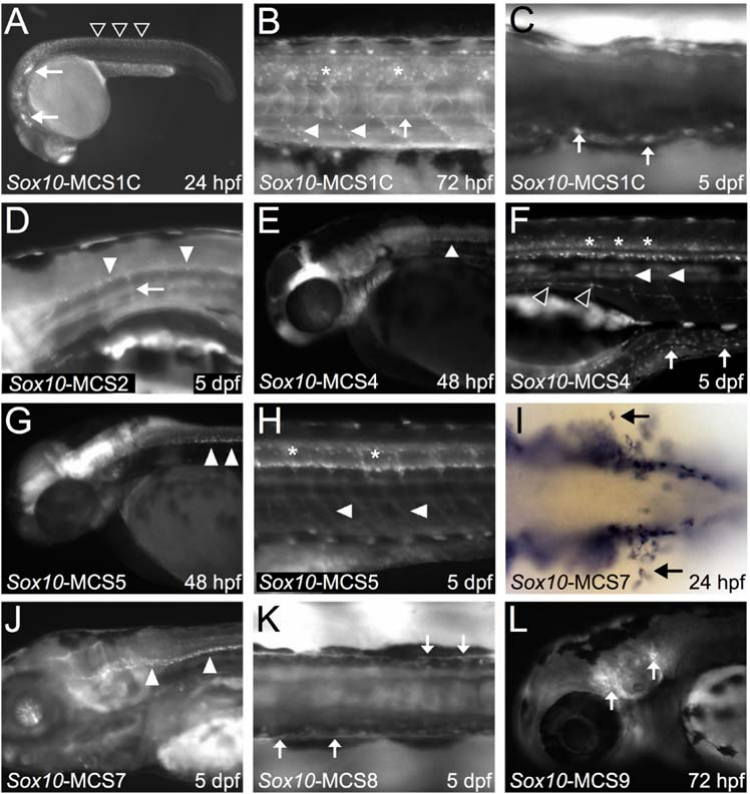
Mouse *Sox10*-MCSs direct EGFP reporter expression in zebrafish embryos consistent with endogenous *sox10*. A–C) *Sox10*-MCS1C directs reporter gene expression to the cranial ganglia (A; white arrows) and within premigratory neural crest (A; open arrowheads) at 24 hpf (A). By 72 hpf (B), signal is detected in scattered oligodendrocytes (asterisks) along the spinal column and descending Schwann cells (white arrowheads) surrounding peripheral motor neurons. Weak reporter expression is also detected along the sympathetic chain (B; white arrow). At 5 dpf (C), signal is clearly detected in the ENS (C; white arrows). D) *Sox10*-MCS2 directs weak EGFP expression in glial cells of the CNS (white arrowheads) and PNS (white arrow). E and F) *Sox10*-MCS4 directs reporter gene expression in presumptive oligodendroglial cells at 48 hpf (E; white arrowhead). By 5 dpf (F), robust signal is detected in several neural crest lineages, including Schwann cells (F; white arrowheads), sympathetic chain ganglia (open arrowheads), and the enteric nervous system (white arrows). As with endogenous *sox10*, EGFP expression is maintained in mature oligodendrocytes (asterisks). G and H) *Sox10*-MSC5 also directs signal in early oligodendroglial cells (G; white arrowheads) and sustains signal in mature oligodendrocytes (asterisks). Glial cells of the PNS (H; white arrowheads) are clearly detected at 5 dpf (H). I) *In situ* hybridization using GFP riboprobe detects early migrating melanoblasts (black arrows) in *Sox10*-MCS7 transgenic embryos at 24 hpf. J) By 5 dpf, *Sox10*-MCS7 directs signal to oligodendrocytes (white arrowheads) along the ventral column. K) *Sox10*-MCS8 directs weak reporter to the ENS (white arrows) at 5 dpf. L) at 72 hpf, sustained reporter gene expression appears in the cranial ganglia (white arrows) of *Sox10*-MCS9 transgenic zebrafish embryos.

The expression of *Sox10* in crest-derived populations and in oligodendrocytes of mammals and fish has been well documented [2],[3],[4],[6],[8],[10],[16],[17],[18],[23]. To determine whether the mouse sequences direct expression in a manner consistent with endogenous *sox10*, we studied the ability of each *Sox10*-MCS to direct reporter gene expression in developing transgenic zebrafish. Importantly, these sequences are not overtly conserved between mammals and teleosts. Furthermore, the limited number of publicly available teleost genomic sequences and the evolutionary distance among them often makes it challenging even to identify sequences conserved within this evolutionary branch (infraclass). Briefly, each mouse segment was cloned upstream of the *c-Fos* minimal promoter and the enhanced green fluorescent protein (EGFP) coding sequence. The resulting constructs were injected into two-cell zebrafish embryos and evaluated for EGFP reporter expression at selected time points (1 to 5 days post-fertilization; dpf). G0 embryos showing consistent reporter gene expression were raised for germline transmission and reporter expression patterns were further evaluated in subsequent generations in two or more founder lines. In agreement with our *in vitro* analyses, neither *Sox10*-MCS1 nor *Sox10*-MCS1B directed reporter gene expression in zebrafish G0 embryos (n = 400; data not shown). By contrast, however, *Sox10*-MCS1C not only directed reporter expression in crest-derived cell lines but also directed tissue-specific reporter expression within neural crest populations *in vivo*, including premigratory neural crest cells, early migrating melanoblasts, cranial ganglia, lateral line ganglia, Schwann cells of the peripheral motor neurons, the sympathetic ganglia, the enteric nervous system, as well as central nervous system oligodendrocytes (Figure 2A through 2C; some data not shown).

**Figure 3 pgen-1000174-g003:**
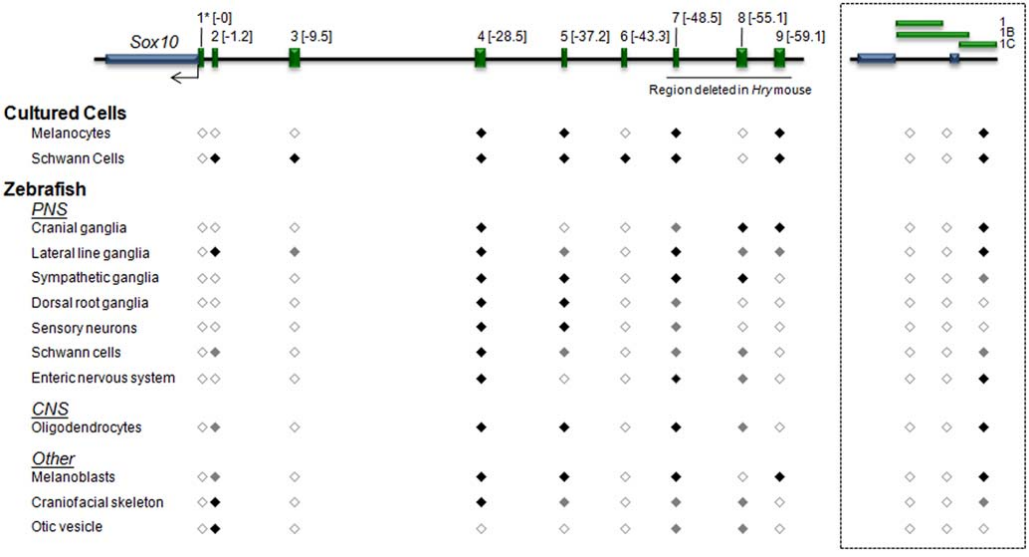
Summary of regulatory activities displayed by *Sox10*-MCSs in cultured cells and in developing zebrafish embryos. A schematic of the assayed *Sox10*-MCS segments is depicted at the top. Distance in kb from the *Sox10* TSS is shown in the brackets adjacent to each construct. Expression in cultured cells and zebrafish cell populations is noted below each construct. For the cultured cells, black-filled diamonds denote at least a 10-fold enhancement in our *in vitro* analysis, while white-filled diamonds fail to reach that threshold. For the neural crest–derived populations and oligodendrocytes evaluated in transgenic zebrafish embryos, black-filled diamonds refer to strong reporter expression, grey-filled diamonds to weaker expression, and white-filled diamonds to an undetected level of reporter expression.

*Sox10*-MCS2 directed expression in neural crest–derived populations of the craniofacial skeleton (data not shown), the otic vesicle (data not shown), developing melanocytes (data not shown), and in glial cells of the peripheral and central nervous systems (Figure 2D). While *Sox10*-MCS3 displayed enhancer activity in cultured Schwann cells, analyses in zebrafish revealed only weak expression in the anterior portion of the lateral line nerve (data not shown). By contrast, and consistent with our *in vitro* data, *Sox10*-MCS4 directed reporter gene expression in a manner that recapitulates endogenous zebrafish *sox10* expression, except for the otic vesicle (Figure 3). Additionally, EGFP expression was also detected in oligodendrocytes at 48 hpf (Figure 2E), as well as in dorsal root ganglia, sympathetic ganglia, and the enteric nervous system at 5 dpf (Figure 2F). *Sox10*-MCS5 also directed EGFP expression in Sox10 positive populations, including the dorsal root ganglia and myelinating glia of both the peripheral and central nervous systems in a manner overlapping temporally with *Sox10*-MCS4 (Figure 2G and H). Interestingly, while *Sox10*-MCS6 directed reporter gene expression in cultured Schwann cells, we did not observe any EGFP expression in zebrafish embryos injected with this construct. Indeed, this was the only identified *in vitro* enhancer that did not drive reporter gene expression in developing zebrafish between 24 hpf and 5 dpf.

The three distal-most assayed segments (*Sox10*-MCS7, *Sox10*-MCS8, and *Sox10*-MCS9) are deleted in the *Sox10^Hry^* mouse model of WS4, which is characterized by distal intestinal aganglionosis and severe hypopigmentation [19]. We hypothesized that these deleted sequences direct expression in affected tissues. Consistent with this hypothesis, *Sox10*-MCS7 directs reporter gene expression in early migrating melanoblasts (Figure 2I) and the enteric nervous system (data not shown). This element also directs expression in dorsal root ganglia and glial cell populations, including oligodendrocytes (Figure 2J) and Schwann cells (data not shown). Although the former cells are not grossly affected in mutant mice, these data are consistent with developmental expression of *Sox10* in mouse [7],[10],[18],[24] and zebrafish [16],[17],[23]. *Sox10*-MCS8 directs reporter gene expression in a subpopulation of the enteric nervous system (Figure 2K), Schwann cells of the peripheral nervous system (data not shown), and the sympathetic ganglia (data not shown). But unlike the other constructs that we assayed, which displayed consistent reporter expression patterns among independent founder lines, *Sox10*-MCS8 showed notable spatial expression differences among multiple lines (n = 4). This was likely a result of position effects. However, while there was variability in expression patterns from the different founders, reporter expression remained limited to sox10 expressing tissues. Moreover, reported sites of expression were consistent among two or more lines. The reporter expression for this construct alone reflects a composite profile from the expression patterns of identified founders. Both *Sox10*-MCS7 and *Sox10*-MCS8 also directed expression in forming otic and craniofacial connective tissue. Finally, *Sox10*-MCS9 directs reporter gene expression in developing melanocytes (data not shown), the lateral line ganglia (data not shown), and cranial ganglia (Figure 2L). Thus, deletion of *Sox10*-MCS7, *Sox10*-MCS8, and *Sox10*-MCS9 likely explain, at least in part, the impaired pigmentation and aganglionic megacolon observed in *Sox10^Hry^* mice.

Interestingly, among the elements driving reporter expression in oligodendrocytes, some direct reporter expression during incompletely overlapping developmental windows. This temporally distinct control is perhaps most notable for sequence elements *Sox10*-MCS5 and *Sox10*-MCS7, as they direct overlapping reporter expression in the forming ventral white matter fiber tracts of the brain and spinal cord at time points between 2 dpf and 5 dpf. Although *Sox10*-MCS5 is detected early (2 dpf) in immature oligodendrocytes (Figure 2G), signal driven by *Sox10*-MCS7 is not readily detected in these populations until more than 24 hours later (data not shown); reporter expression driven by this sequence ultimately then overlaps with persistent expression driven by *Sox10*-MCS5 by 5 dpf (Figure 2H and J). These observations highlight the power of temporally dynamic transgenic analyses in zebrafish, as myelination in mammals is a post-natal event and would escape mid-gestational analyses.

### *Sox10*-MCSs Direct Reporter Gene Expression in a Manner Consistent with *Sox10* in Transgenic Mice

Our analyses represent the first report of mammalian non-coding sequences at *Sox10* directing reporter gene expression in zebrafish and display success consistent with recent analyses of the *RET* locus. To determine the fidelity of these observations, we assayed a subset of the identified enhancers in mice, prioritizing two genomic segments whose regulatory control can independently account for a significant fraction of, if not all, *Sox10* expression. First, *Sox10*-MCS4 modulated expression in nearly every *sox10* expressing structure in zebrafish. Second, *Sox10*-MCS7, which is deleted in the *Sox10^Hry^* mouse model of WS4 [19], directed similarly broad *sox10*-appropriate expression, including in developing melanocytes and the ENS. We therefore examined the ability of *Sox10*-MCS4 and *Sox10*-MCS7 to direct reporter gene expression in developing mouse embryos.

**Figure 4 pgen-1000174-g004:**
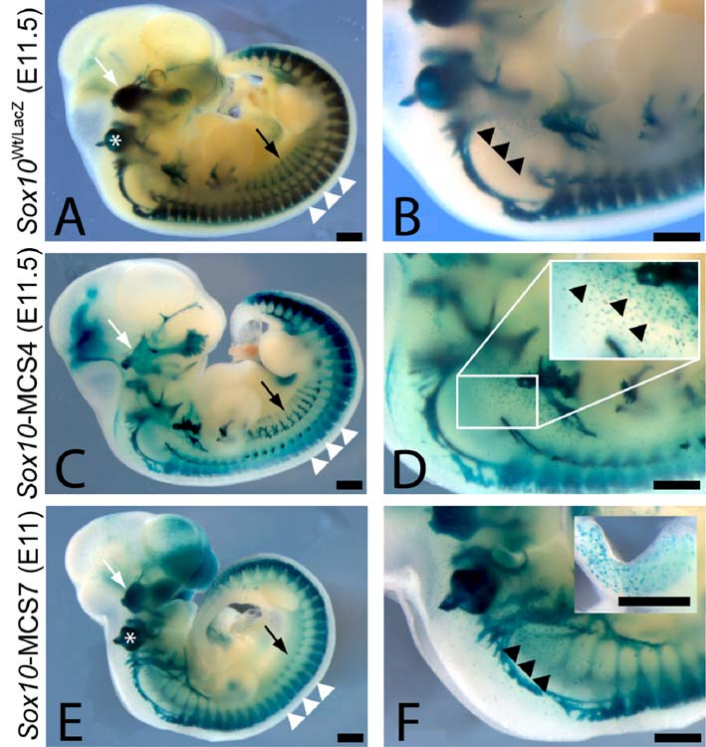
*Sox10*-MCS4 and *Sox10*-MCS7 direct reporter expression in developing transgenic mice consistent with their activity in zebrafish. LacZ reporter expression was detected at the resolution of whole-mount for *Sox10* ^Wt/tm1Weg^ embryos at E11.5 (A and B), *Sox10*-MCS4 embryos at E11.5 (C and D), and *Sox10*-MCS7 embryos at E11 (E and F). The broad expression of *Sox10* in neural crest–derived populations is apparent in *Sox10* ^Wt/tm1Weg^ embryos (A and B), including the cranial ganglia (white arrow), otic vesicle (asterisk), sympathetic ganglia (black arrow), dorsal root ganglia (white arrowheads), and melanoblasts (black arrowheads). Both *Sox10*-MCS4 (C and D) and *Sox10*-MCS7 (E and F) also direct similarly broad reporter expression in these neural crest tissues. D and F insets represent melanoblast and enteric ganglia expression, respectively. Scale bar = 500 µm.

Briefly, each genomic segment was cloned upstream of a minimal promoter (*Hsp68*) and *LacZ* coding sequence, and the resulting constructs were injected into mouse pronuclei. Reporter gene expression was then studied at specific developmental time points (E11.5 and E13.5), and compared to the known expression of *Sox10*, via a *LacZ* reporter gene inserted at the *Sox10* locus [18] (Figure 4A and B). *Sox10*-MCS4 directed reporter gene expression in nearly every tissue in which *Sox10* is expressed at E11.5 (n = 2), at the resolution of whole mount embryo analysis (Figure 4C and D). Consistent with our observations in zebrafish, we detected reporter gene expression in the cranial, dorsal root and sympathetic ganglia (Figure 4C), the enteric nervous system (data not shown), and in melanoblasts (Figure 4D, inset). The only relevant structure in which reporter gene expression was not observed was the otic vesicle, consistent with our observations in zebrafish. Similarly, at E13.5 reporter expression directed by *Sox10*-MCS4 provided a near complete recapitulation of the endogenous *Sox10* gene product (n = 3); for example, expression was observed in sensory neurons, sympathetic ganglia, the enteric nervous system, and melanoblasts (data not shown). Given the absence of sequence conservation between the corresponding sequences from these species, data resulting from the analysis of the mouse *Sox10*-MCS4 sequence in mice and fish display a remarkable concordance.

*Sox10*-MCS7 likewise directed reporter gene expression consistent with both endogenous *Sox10* expression at E11.5 (n = 1) and with our analyses in transgenic zebrafish. *LacZ* expression was detected in the cranial ganglia (Figure 4E), dorsal root ganglia (Figure 4E), otic vesicle (Figure 4E), melanoblasts (Figure 4F), sympathetic ganglia (Figure 4E), and in the enteric nervous system (Figure 4F, inset). These data underscore the potential role for *Sox10*-MCS7, among others, in the developmental regulation of *Sox10* transcription. In particular, they suggest that this segment plays a role in *Sox10* expression during melanocyte migration and development, and support the hypothesis that deletion of this element may contribute to the impaired pigmentation observed in the *Sox10^Hry^* mouse model of WS4 (see above). Furthermore, *Sox10*-MCS7 also directs expression in the mouse enteric nervous system, suggesting that deletion of this region may also contribute to the aganglionic megacolon observed in *Sox10^Hry^* mice. Taken together, these data suggest that analysis of mouse *Sox10* regulatory sequences in zebrafish serves as a reliable surrogate for their analysis in mice. These studies revealed two enhancers with near pan-neural crest regulatory control, including sequences directing expression in melanoblasts.

### Targeted Mutagenesis of SoxE Consensus Sequences within *Sox10*-MCS4 and *Sox10*-MCS7 Dramatically Alters Enhancer Activity

**Figure 5 pgen-1000174-g005:**
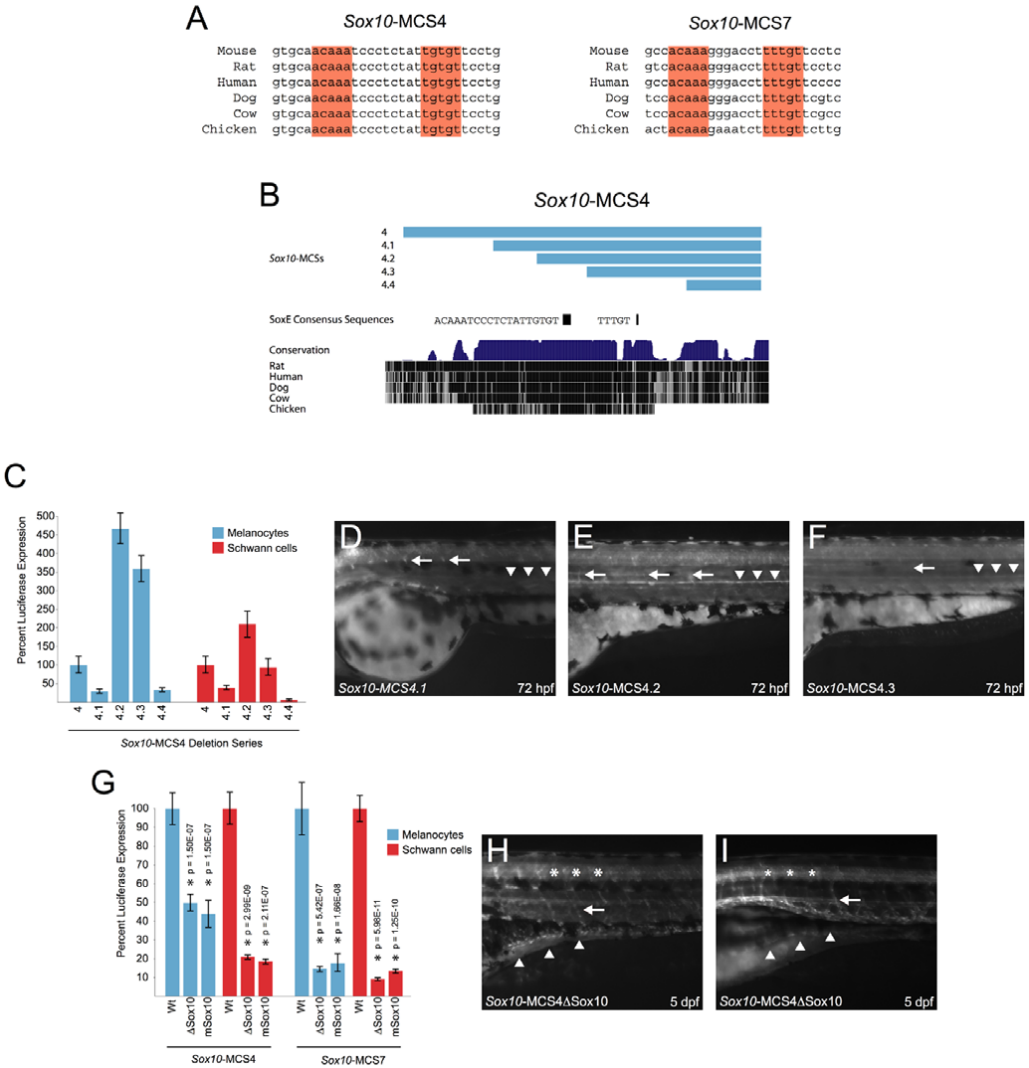
Analyses of a dimeric SoxE consensus sequence within *Sox10*-MCS4 and *Sox10*-MCS7. A) Consensus SoxE family binding sites are oriented in a head-to-head fashion within *Sox10*-MCS4 and *Sox10*-MCS7. B) A deletion series across *Sox10*-MCS4 (pale blue bars) is depicted in the UCSC Genome Browser. The position and sequence of a monomeric and a dimeric SoxE consensus sequence is shown below the deletion series. C) *In vitro* enhancer activity for each fragment of the deletion series was tested individually in melan-a cells (blue bars) and S16 cells (red bars). The results for *Sox10*-MCS4 are included for cross-comparison of modular enhancer activity. Error bars, SD. D–F) *in vivo* enhancer activity was compared at 72 hpf for *Sox10*-MCS4.1 through *Sox10*-MCS4.3 in transgenic zebrafish embryos. *Sox10*-MCS4.4 failed to direct reporter expression in G0 embryos and was not raised for germline transmission. *Sox10*-MCS4.1 (D) directed reporter expression to Schwann cells (white arrows) and weak reporter expression to sympathetic ganglia (white arrowheads). *Sox10*-MCS4.2 (E) directed reporter expression in an opposite fashion, as signal appeared weaker in Schwann cells and more robust in sympathetic ganglia. *Sox10*-MCS4.3 (F) directed an extremely low level of reporter expression to these two neural crest–derived populations, and the arrow and arrowheads show the relative position of where the Schwann cells and sympathetic ganglia is normally positioned. G) Site-directed mutagenesis was used to delete and mutate the dimeric SoxE consensus sequence within *Sox10*-MCS4 and *Sox10*-MCS7. These mutagenized constructs were tested for *in vitro* enhancer activity in melanocytes (blue bars) and Schwann cells (red bars), and compared against their wild-type sequences. P-values are given above each tested construct and error bars indicate the standard deviation. H and I) *Sox10*-MCS4 with a deleted head-to-head SoxE family binding site was selected for transmission through the germline. Analysis of two different founder lines (H and I, respectively) revealed a decrease in signal in oligodendrocytes (asterisks) and scattered reporter expression in a subset of the ENS (white arrowheads). Schwann cells (white arrow), however, appear to be unaffected.

Members of the SoxE transcription factor family (Sox8, Sox9, and Sox10) bind DNA as either monomers, wherein they bind to single SRY-like consensus sequences (e.g., ACAAA), or as dimers that bind to two SRY-like binding sites oriented in a head-to-head fashion (hereafter referred to as dimeric SoxE consensus sequences) [25]. Our previous computational and functional analyses revealed highly-conserved dimeric SoxE consensus sequences in *Sox10*-MCS7 [19]. Furthermore, it has previously been hypothesized that SoxE proteins (e.g., SOX9) directly regulate the transcription of SOX10 [26]. To determine if SoxE proteins might regulate other genomic segments at *Sox10*, we searched each *Sox10*-MCS for similar consensus sequences. Interestingly, both *Sox10*-MCS4 and *Sox10*-MCS7 contain dimeric SoxE consensus sequences that are perfectly conserved between mammals and chicken (Figure 5A), consistent with their demonstrated biological functions and with the suggestion that these sequences have an important role in the transcriptional regulation of *Sox10*.

To test this hypothesis, we established a deletion series across *Sox10*-MCS4 (Figure 5B) to identify sequence intervals that are essential for aspects of its regulatory control. We assayed each resulting genomic segment for the ability to direct reporter gene expression *in vitro* and *in vivo* as before. In cultured melanocytes and Schwann cells, the assayed constructs modulated reporter gene expression with each deletion (Figure 5C). Specifically, removal of 5′ sequences to create *Sox10*-MCS4.1 caused a ∼75% and ∼60% reduction of enhancer activity in melanocytes and Schwann cells, respectively. By contrast, removal of additional 5′ sequences to create *Sox10*-MCS4.2 resulted in a ∼4.5-fold and ∼2-fold increase in enhancer activity compared to *Sox10*-MCS4 in melanocytes and Schwann cells, respectively. This significant increase in activity may result from the removal of sequences adjacent to the dimeric SoxE consensus sequences in *Sox10*-MCS4.2 (Figure 5B). This observation is consistent with analysis of *Sox10*-MCS7, wherein *in vitro* activity is increased upon the deletion of sequences flanking a core 95-basepair fragment containing the dimeric SoxE consensus sequences [19]. Further deletion of sequences from the 5′ end of *Sox10*-MCS4.2 to create *Sox10*-MCS4.3 reduced enhancer activity below levels observed for *Sox10*-MCS4.2 in both cell lines. Interestingly, this deletion removes the dimeric SoxE consensus sequence, but does not disrupt a highly conserved, monomeric SoxE consensus sequence (Figure 5B), suggesting that the interval containing the dimeric sequence contributes significantly to the observed activity. The remaining SoxE consensus sequence may also explain the less dramatic effect of deleting the dimeric SoxE consensus sequences in *Sox10*-MCS4 compared to *Sox10*-MCS7 (see below). Finally, the removal of sequences encompassing this consensus sequence to create *Sox10*-MCS4.4 severely reduced enhancer activity below that of *Sox10*-MCS4, especially in cultured melanocytes (Figure 5C). These data suggest that the intervals containing SoxE binding sites play a critical role in the activation of reporter expression controlled by *Sox10*-MCS4 and *Sox10*-MCS7 in these cell lines. We thus directly assayed their necessity through independent deletion and mutation of the dimeric SoxE consensus sequences in both genomic segments (Figure 5G) and compared the ability of each wild-type and mutated genomic segment to direct reporter gene expression in the above cell lines. Consistent with this postulate, deletion or mutation of the dimeric SoxE consensus sequences significantly reduced the enhancer activity in both cell lines (Figure 5G; p-values between 5.98×10^−11^ and 2.11×10^−7^). This effect was highly significant for both deletion and mutation of these sequences within both elements, albeit more severe for *Sox10*-MCS7 (≥80% reduction in enhancer activity, p≤5.42×10^−7^) than for *Sox10*-MCS4 (50–80% reduction in activity, p≤2.11×10^−7^). Together these data support the hypothesis that the dimeric SoxE consensus sequences are important for the function of both genomic segments in these two cell types (see above). They further suggest that although *Sox10*-MCS7 appears to be highly dependent on the dimeric SoxE consensus sequences for its function, domains in addition to this motif also contribute aspects of the biological potential demonstrated by *Sox10*-MCS4.

To determine the *in vivo* functional requirement for each of the *Sox10*-MCS4 sequence intervals described above, including the dimeric SoxE sequences, we studied each *Sox10*-MCS4 fragment (wild type and mutant) in transgenic zebrafish embryos. Once again all constructs were passed through the germline and evaluated in multiple founder lines to ensure the integrity of our analyses. *Sox10*-MCS4.1 directed expression in a similar manner to that observed for *Sox10*-MCS4, however with significantly reduced intensity. For example, low levels of expression were observed in Schwann cells surrounding spinal motor neurons, and almost no signal was observed in the sympathetic ganglia (Figure 5D). In contrast, *Sox10*-MCS4.2 directed reporter gene expression at higher levels in sympathetic chain ganglia and at reduced levels in glia surrounding spinal motor neurons when compared to *Sox10*-MCS4.1 (Figure 5E). Interestingly, this observed flux in signal intensity is completely consistent with our *in vitro* observations. A more dramatic loss of reporter gene expression was evident upon analysis of *Sox10*-MCS4.3; expression was severely reduced in populations such as the cranial and sympathetic ganglia at 24 hpf (data not shown), as well as in oligodendrocytes and Schwann cells at 72 hpf (Figure 5F). This is similarly consistent with our *in vitro* observations and with the hypothesis that the dimeric SoxE motif is important for *Sox10*-MCS4 function. Finally, injection of over 400 G0 zebrafish embryos with *Sox10*-MCS4.4 failed to direct detectable reporter gene expression in any structures, indicating that all sequences necessary to direct tissue-specific reporter gene expression had been deleted. These data are also consistent with our *in vitro* analyses and may be explained by the removal of the monomeric SoxE consensus sequence (see above).

To determine whether the observed reporter deficits in fish injected with *Sox10*-MCS4.3 were the result of removing the dimeric SoxE consensus sequences, we next evaluated transgene reporter expression directed by *Sox10*-MCS4 harboring a deletion of the dimeric consensus sequences alone. The resulting embryos displayed a decrease in EGFP-positive enteric ganglia (compare Figure 5H and I to Figure 2F); reporter gene expression was only detected in dorsally located ganglia, suggesting that the consensus sequence may be critical for a subset of cells in the enteric nervous system. Expression was also severely reduced in oligodendrocytes (compare Figure 5H and I to Figure 2E and F). Interestingly, expression in Schwann cells was not disrupted (compare Figure 5H and I to Figure 2F). These data were consistent upon analysis of at least two independent founder lines (Figure 5H and I) and suggest that the dimeric SoxE consensus sequences in *Sox10*-MCS4 play a more critical role regulating *Sox10* expression during oligodendrocyte and ENS development than in Schwann cell development at the evaluated time points.

### Identification of the Zebrafish Functional Orthologs of *Sox10*-MCS4 and *Sox10*-MCS7

**Figure 6:**
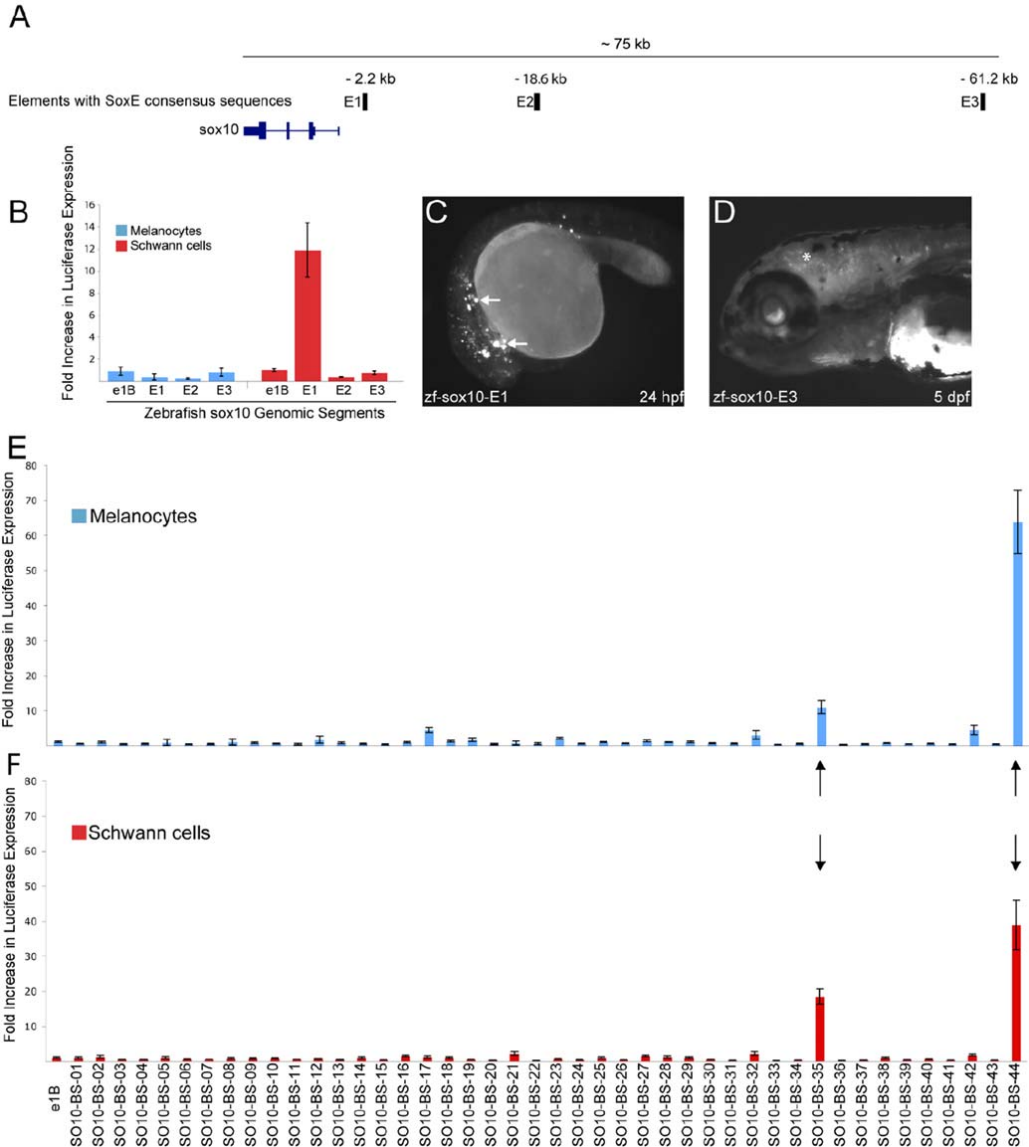
Motif-based search for functional zebrafish orthologs and SoxE positive enhancers. A) A computational search for dimeric SoxE consensus sequences in a 100 kb window upstream of the zebrafish *sox10* gene resulted in three hits (pale blue bars). The distance from the *sox10* TSS is denoted above each enhancer (E1–E3). B) A 500 bp region surrounding each dimeric SoxE consensus sequence was tested for *in vitro* enhancer activity in a melanocyte cell line (blue bars) and a Schwann cell line (red bars). Each fragment is compared against a control (pE1b with no insert) and error bars indicate the standard deviation. C) zf-*sox10*-E1 directs reporter expression in scattered cells throughout the 24 hpf, G0 embryo, consistent with migrating neural crest cells (white arrows). D) zf-*sox10*-E3 directs reporter expression in cranial oligodendrocytes (asterisks) at 5 dpf in G0 embryos. E and F) Data mining of the mouse genome for highly conserved dimeric SoxE consensus sequences in the intron of genes expressed in melanocytes identified 44 genomic segments. *In vivo* enhancer activity was tested in cultured melanocytes (E) and Schwann cells (F). Only two segments drove luciferase expression above a 10-fold threshold in both cell lines (black arrows). Error bars, SD.

We have shown that highly conserved mouse sequences at *Sox10* direct tissue-specific reporter gene expression in developing transgenic zebrafish. These observations belie the fact that there is no observable non-coding sequence conservation between mammals and zebrafish at *Sox10* (Figure 1A), and are consistent with previous analyses at the *RET* locus [27]. To identify the zebrafish orthologs of *Sox10*-MCS4 and *Sox10*-MCS7, we exploited the observation that these two genomic segments harbor highly conserved dimeric SoxE consensus sequences. Briefly, we computationally identified all dimeric SoxE consensus sequences in a 100 kb window upstream of the zebrafish *sox10* locus. These analyses identified three such consensus sequences (Figure 6A). We hypothesized that sequences encompassing these motifs would function as *in vitro* and *in vivo* enhancers in a manner overlapping *Sox10*-MCS4 and/or *Sox10*-MCS7. Thus, we PCR amplified 500 bp surrounding each of these consensus sequences (zf-*sox10*-E1, zf-*sox10*-E2, and zf-*sox10*-E3; Figure 6A and Table 1) from zebrafish genomic DNA and tested each *in vitro* and *in vivo* as above. zf-*sox10*-E1 displays strong enhancer activity in Schwann cells, directing reporter gene expression ∼12-fold higher than the control vector (Figure 6B). In contrast, neither zf-*sox10*-E2 nor zf-*sox10*-E3 displays strong enhancer activity in either cell line. However, it is important to note that these cells do not fully represent the range of cell types or stages in cell maturation in which *sox10* is expressed during development. Indeed, when assayed *in vivo*, both zf-*sox10*-E1 and zf-*sox10*-E3 but not zf-*sox10*-E2 direct EGFP expression in cell populations consistent with endogenous *sox10* expression. zf-*sox10*-E1 directed reporter expression in scattered cells in zebrafish G0 embryos (24 hpf) in a manner consistent with migrating neural crest, appearing to be positioned at multiple points along the anterior posterior axis both dorso-laterally and along a ventral path (Figure 6C). Additionally, although zf-*sox10*-E3 did not display enhancer activity in Schwann and melanocyte cell lines, it did direct reporter gene expression in forming oligodendrocytes of the central nervous system (Figure 6D) of mosaic G0 embryos. Importantly, these observations underscore the benefit of evaluating regulatory control within an intact organism.

Taken together, the presence of such a small number of conserved dimeric SoxE consensus sequences in the evaluated interval and the data from *in vitro* and *in vivo* functional assays strongly suggest that zf-*sox10*-E1 and zf-*sox10*-E3 are the zebrafish orthologs of *Sox10*-MCS4 and *Sox10*-MCS7, respectively. This is further supported by the relative position of each genomic segment in the mouse and zebrafish genomes (compare Figure 1A to [Fig pgen-1000174-g002] [Fig pgen-1000174-g003] [Fig pgen-1000174-g004] [Fig pgen-1000174-g005] 6A) and their associated regulatory control. However, one might expect that the simple identification of conserved dimeric SoxE consensus sequences would yield a high number of elements with *in vitro* enhancer activity, and that these data may reflect a high false positive rate. However, note that zf-*sox10*-E2 did not direct reporter gene expression in cultured cells (Figure 6B) or developing zebrafish (data not shown). To determine the frequency with which sequences encompassing a highly conserved SoxE consensus sequence will identify enhancers that function in a subset of SoxE positive tissues, we computationally identified all dimeric SoxE consensus sequences in the mouse genome. We then identified the subset of these consensus sequences that have a conservation profile consistent with *Sox10*-MCS4 and *Sox10*-MCS7 and that reside within intronic regions of genes expressed in melanocytes (see methods for details). We chose to examine intronic sequences because: (1) they provide a definable genomic element in which to search; (2) introns are known to frequently harbor enhancer sequences; and (3) functional intronic SoxE binding sites have been identified within introns of other SOX10 target genes [e.g., myelin protein zero (MPZ) [28]]. These analyses provided a dataset of 44 genomic segments (Table S1), which were then tested for enhancer activity in cultured melanocytes and Schwann cells as described above. Only two of the 44 genomic segments (4.5%) increased reporter gene expression at least 10-fold higher in either cell line; both directed reporter gene expression in cultured melanocytes (Figure 6E) and Schwann cells (Figure 6F), suggesting they may be true SoxE target enhancers. Although we anticipate that some degree of the low frequency of success may arise from the amplification and assay of incomplete regulatory modules or from their assay in inappropriate crest derivatives, we anticipate that many are simply false negatives. Thus, our data suggest that implementing this approach at orthologous loci may enrich for correctly identified crest enhancers, and on a genomic scale, greater information regarding the composition and configuration of critical binding site motifs will be necessary.

## Discussion

*SOX10* encodes a developmentally critical transcription factor whose roles in development and disease have been well described. Mutations in *SOX10* underlie defects in several neural crest–derived populations, resulting in epidermal hypopigmentation, enteric aganglionosis, and demyelinating peripheral neuropathy; outside neural crest–derived populations, they can also result in dysmyelinating pathologies of the central nervous system oligodendrocytes [11],[13],[14],[15]. The pleiotropic roles of *Sox10* are facilitated by tightly controlled and dynamic regulatory control, encoded by sequences at this locus but until recently, little was known of the cis-acting sequences that modulate its dynamic expression in vertebrates [29],[30]. The first step in deciphering this regulatory lexicon is the identification of cis-acting regulatory sequences within and flanking *SOX10*. We directly addressed this challenge, assaying the regulatory control exerted by highly conserved, non-coding sequences at the mouse *Sox10* locus in cultured cells and *in vivo* through transgenesis in zebrafish and mice.

Data generated *in vitro* and *in vivo* was highly concordant, and critically identified regulatory elements were consistent with all aspects of *Sox10* expression during embryogenesis, in both NC-derived and non-NC-derived structures. For example, six genomic segments (*Sox10*-MCS1C, *Sox10*-MCS2, *Sox10*-MCS4, *Sox10*-MCS5, *Sox10*-MCS7, and *Sox10*-MCS9) directed EGFP expression in developing melanocytes, and six genomic segments (*Sox10*-MCS1C, *Sox10*-MCS2, *Sox10*-MCS4, *Sox10*-MCS5, *Sox10*-MCS7, and *Sox10*-MCS8) directed EGFP expression in Schwann cells during zebrafish embryogenesis. These are the first data showing that non-coding sequences at *Sox10* direct expression in these NC derivatives. Furthermore, six genomic segments (*Sox10*-MCS1C, *Sox10*-MCS2, *Sox10*-MCS4, *Sox10*-MCS5, *Sox10*-MCS7, and *Sox10*-MCS8) directed EGFP expression in oligodendrocytes; these represent the first data showing that non-coding sequences at *Sox10* direct expression in this non-NC-derived cell population. Importantly, these data were generated in teleosts using mouse genomic sequence with which we detected no overt sequence conservation.

In an effort to evaluate the fidelity of our observations in zebrafish, we likewise assayed two genomic segments using transgenesis in developing mice, prioritizing sequences that displayed broad *Sox10*-appropriate regulatory control in teleosts (*Sox10*-MCS4 and *Sox10*-MCS7). The resulting data strongly suggest that zebrafish transgenesis serves as a reliable surrogate for the assayed sequences; *Sox10*-MCS4 and *Sox10*-MCS7 both directed reporter gene expression in nearly all endogenous sites of *Sox10* expression during key stages of mouse development (Figure 4). Taken together, one of the most striking observations is the degree of functional overlap shared among identified regulatory sequences. This appears to be an increasingly common observation at developmental genes [31],[32],[33],[34] and suggests a degree of functional redundancy in their regulation. However, in this case, we know that this is unlikely; our recent *Sox10^Hry^* mouse model of Waardenburg-Shah Syndrome demonstrated that homozygous mutant mice harboring carry a deletion that encompasses *Sox10*-MCS7, *Sox10*-MCS8, and *Sox10*-MCS9 display severe hypopigmentation and enteric aganglionosis. Data presented in this study strongly suggests that the deletion of these three regulatory elements contribute to the enteric and pigmentary phenotype of the *Sox10^Hry^* mice [19]. However, these are not the only regulatory elements contributing to expression in the affected cell types; *Sox10*-MCS1C, *Sox10*-MCS4, and *Sox10*-MCS5, among others, are not deleted in the *Sox10^Hry^* mice yet they also direct reporter gene expression in developing melanocytes and/or the enteric nervous system (Figure 3). Thus, a parsimonious explanation for the observed data is that multiple regulatory sequences are coordinately required for the proper spatial and temporal expression of *Sox10* during development, each contributing to expression in certain subsets of tissues in an additive or interactive manner. To fully address this issue, it will be necessary to perform cell- and tissue-specific ChIP, as well as deletion and mutation analysis of each *Sox10*-MCS within the context of the entire gene (e.g., in BAC transgenic mice).

Consistent with our data, Werner et al. [30] made similar observations in their preliminary evaluation of a small number of overlapping fragments in mice. However, there are a number of notable discrepancies, especially when considering regulatory control in developing melanocytes, sympathetic ganglia and glial cells. First, our analyses revealed six genomic segments (*Sox10*-MCS1C, *Sox10*-MCS2, *Sox10*-MCS4, *Sox10*-MCS5, *Sox10*-MCS7, and *Sox10*-MCS9) that direct reporter gene expression in developing melanocytes, consistent with the known role of *Sox10* in these cells. Werner and colleagues [30] assayed three genomic segments that partially overlap with sequences that we assayed. (Their sequences, termed U1, U2, and U3 correspond to *Sox10*-MCS7, *Sox10*-MCS5 and *Sox10*-MCS4 respectively). None of the “U” elements directed reporter gene expression in melanoblasts, including U1 and U3, which correspond to *Sox10*-MCS7 and *Sox10*-MCS4, respectively. However, our analyses of these genomic segments in developing mouse embryos revealed clear expression in melanoblasts of mice at E11, E11.5 and E13.5 (Figure 4 and data not shown), consistent with our observations in transgenic zebrafish embryos (Figure 2). Importantly, U3 lacks 170 bp present in *Sox10*-MCS4, suggesting that the additional sequences in *Sox10*-MCS4 may be important for expression in developing melanocytes. In contrast, U1 and U2 both encompass and are 38% and 49% larger than *Sox10*-MCS7 and *Sox10*-MCS5, respectively, yet in our assays both genomic segments directed reporter gene expression in developing melanocytes; importantly, in the case of *Sox10*-MCS7, this was substantiated by reporter expression in melanocyte cell lines as well as reporter expression in melanoblasts of transgenic zebrafish and mouse embryos. One possible explanation is that these U elements also harbor local repressor sequences that are active in developing melanocytes. Another possibility is that position-related effects altered the activity of U1, U2, and U3 such that expression in melanoblasts was not detectable. Importantly, our data strongly suggest that *Sox10*-MCS4 and *Sox10*-MCS7 are important for expression during mammalian melanocyte development, and represent the first report of transcriptional regulatory elements important for *Sox10* expression in melanoblasts.

Second, similarly in our evaluation of *Sox10*-MCS4 in transgenic zebrafish and mouse, we observed expression in the sympathetic ganglia. Once again, this contrasts with observations for U3 [30]. Interestingly, U3 ceases sequence overlap with *Sox10*-MCS4 at a point where our assayed deletion fragment *Sox10*-MCS4.4 begins; critical motifs contained within this fragment may work in conjunction with other transcription factor binding sites found throughout the *Sox10*-MCS4 sequence but are not sufficient to independently direct reporter gene expression to the sympathetic ganglia. Another possibility is that U3 harbors a local repressor within the 32 bp that it extends beyond our *Sox10*-MCS4 element.

Third, *Sox10* expression in developing oligodendrocytes and Schwann cells of the central and peripheral nervous system, respectively, is critical for the formation of all white matter fiber tracts [6],[7],[9],[10],[18]. Indeed, six of the genomic segments at *Sox10* analyzed in zebrafish revealed expression in oligodendrocytes and Schwann cells (*Sox10*-MCS1C, *Sox10*-MCS2, *Sox10*-MCS4, *Sox10*-MCS5, *Sox10*-MCS7, and *Sox10*-MCS8). However, although U1-3 overlap *Sox10*-MCS4-7 as described above, the authors were unable to detect reporter expression in these populations because myelination is a post-natal developmental event in mammals [6],[8],[10]. By contrast, myelination in zebrafish begins at 2–3 days post fertilization in zebrafish and thus, the *in vivo* enhancer activity of genomic segments important for expression in myelinating cells can be readily detected. Our data underscore another major strength of employing zebrafish to study the transcriptional regulation of *Sox10*. Indeed, this is the first report of transcriptional regulatory elements important for *Sox10* expression in Schwann cells and oligodendrocytes. Additionally, we demonstrate that the same elements exert regulatory control in both populations.

Additionally, in a recent report of BAC-based transgenic rescue of *Sox10* deficient mouse phenotypes, Deal and colleagues [29] report the evaluation of a BAC transgene termed DelnA, wherein most sequence 5′ to the *Sox10* coding sequence has been deleted. Although, this BAC appears to retain the regulatory sequences we have termed *Sox10*-MCS1C and *Sox10*-MCS2, it directs reporter expression only in the otic canals and melanoblasts, and weakly in the superior cervical ganglia. Given our observation that when assayed independently these sequences direct expression in many neural crest derivatives, one parsimonious explanation is that, in context, these sequences must behave coordinately and/or overcome local repression in order to elicit their requisite control. Taken in combination, these observations may explain previous failures to generate effective *Sox10* reporter transgenes using conventional “promoter bashing” strategies. Consequently, *Sox10*-MCS1C may perhaps be more readily considered a proximal enhancer than a regulatory promoter of *Sox10*.

Our computational and functional analyses of conserved, non-coding sequences at *Sox10* have illuminated the potentially important role of SoxE regulation of *Sox10*. We have previously demonstrated that deletion of a large region harboring a dimeric SoxE consensus sequence in mouse gives rise to a phenotype similar to *Sox10* coding mutations [19]. Here, we also report that two genomic segments (*Sox10*-MCS4 and *Sox10*-MCS7) harbor highly-conserved dimeric SoxE consensus sequences that are essential for the appropriate control of reporter gene expression in relevant cells and tissues. However, while the dimeric SoxE consensus sequences analyzed in this study are likely important for *Sox10* expression, our data also indicate that they are not essential for all regulatory control exerted by the sequences containing them and, indeed, other sequences within these segments are likely to be similarly important. Consistent with this idea we also provide deletion of sequences upstream of the SoxE consensus sequences in *Sox10*-MCS4 also significantly reduce reporter gene expression (*Sox10*-MCS4.1, Figure 5C). Thus, it is likely that other sequences within the studied genomic segments also contribute to the appropriate regulatory control of *Sox10*. Elucidation of this underlying vocabulary will be critical for a full understanding of *Sox10* expression during vertebrate development.

Although we successfully implemented the zebrafish transgenic assays using mouse genomic DNA with conservation restricted to mammalian and avian species, the absence of corresponding sequence conservation between mammals and fish prevented us from directly identifying the zebrafish orthologs of each mouse *Sox10*-MCS. We were able, however, to uncover potential zebrafish orthologs of *Sox10*-MCS4 and *Sox10*-MCS7 by cataloging dimeric SoxE consensus sequences upstream of the zebrafish *sox10* gene (n = 3). Of the three fragments that harbor these consensus sequences, two (zf-*sox10*-E1 and zf-*sox10*-E3) directed reporter gene expression in *Sox10* relevant cells and tissues. Furthermore, we demonstrated that the likelihood of stumbling upon a *Sox10*-like regulatory element by chance based only on the presence of dimeric SoxE consensus sequences was very low; only two of 44 genomic segments that harbor such sequences and that reside within non-coding sequences at genes expressed in melanocytes directed reporter expression in a corresponding cell line. Thus, we propose that zf-*sox10*-E1 and zf-*sox10*-E3 are the zebrafish orthologs of *Sox10*-MCS4 and *Sox10*-MCS7, respectively. These observations suggest that motif-based strategies may be useful for identifying the functional orthologs of cis-acting transcriptional regulatory elements in the absence of overt non-coding sequence conservation.

In characterizing *Sox10*-MCS4 and *Sox10*-MCS7, we have described the first pan-neural crest enhancers; these genomic segments direct reporter gene expression in nearly all tissues in which *Sox10* is expressed, including peripheral nervous system neurons, developing melanocytes, and glial cells. As such, the constructs and transgenic zebrafish strains we have developed provide critical tools for studying the role of *Sox10*, and more generally, the neural crest in human development and disease. Additionally, these genomic segments will now be scrutinized for variants that may cause or convey susceptibility to disease.

The studies reported here further our knowledge of the transcriptional regulation of the *Sox10* locus and underscore the complex regulatory control of this developmentally critical transcription factor. Specifically, we make several notable observations. First, multiple genomic segments direct reporter gene expression in overlapping neural crest derivates and glial cells. Second, at least two genomic segments direct expression in a pan-neural crest manner and SoxE consensus sequences are required for the integrity of their regulatory control. Third, genomic sequences derived from the mouse *Sox10* locus direct appropriate reporter gene expression in developing zebrafish, even in the absence of observable conservation and demonstrate that zebrafish provide a high fidelity surrogate for the evaluation of mammalian *Sox10* regulatory sequences. Finally, we provide the first report of *Sox10* regulatory sequences that direct expression in developing melanocytes and glia. These findings have important implications for studying neural crest development and the etiology of related diseases, and, more generally, provide a paradigm for dissecting the regulatory control of genes implicated in human disease.

## Methods

### Vector Construction

Expression constructs were generated using Gateway technology (Invitrogen, Carlsbad, CA). Briefly, PCR primers containing flanking attB sites were designed to amplify each genomic region of interest. Subsequent to PCR reactions, purified products were recombined into the pDONR221 vector according to the manufacturer's specifications (Invitrogen). Each insert was sequenced to ensure the absence of PCR-induced errors. Mutagenesis of MCS4 and MCS7 was performed using the QuikChange Site-Directed Mutagenesis Kit (Stratagene, La Jolla, CA) and appropriate mutation-bearing oligonucleotides. Subsequently, plasmid DNA isolated from each entry clone was recombined with either pLGF-E1b (for luciferase reporter gene expression in culture cells) [19], pT2cfosGW (for EGFP expression in developing zebrafish embryos) [27], or Hsp68-LacZ (for *LacZ* expression in developing mouse embryos) [35] destination vectors according to the manufacturer's specifications (Invitrogen). Each resulting construct underwent restriction enzyme digest with BsrGI, EcoRV, or SalI, respectively, to confirm the presence of the appropriately sized insert.

### Cell Culture, Transfections, and Luciferase Activity Assays

Immortalized melanocytes (melan-a) [36], Schwann cells (S16) [37], and NIH-3T3 cells were cultured under standard conditions. 5×10^4^ cells were placed into each well of a 96-well culture plate and transfected with luciferase reporter vectors (see above) using lipofectamine 2000 reagent (Invitrogen) according to the manufacturer's instructions. For each reaction, 0.25 µl of lipofectamine 2000 and 25 µl of OptiMEM I minimal growth medium (Invitrogen) were combined and incubated at room temperature for 10 minutes. Purified luciferase reporter vector (200 ng) or the equivalent volume of water (in the case of DNA-negative controls), and 2 ng of the internal control pCMV-RL renilla expression vector (Promega, Madison, WI) were diluted in 25 µl of OptiMEM I and combined with the lipofectamine-OptiMEM I mixture. The ∼50 µl reactions were incubated at room temperature for 20 minutes and then added to a single well of the 96-well culture plate containing cells. After a 3 hour incubation at 37°C, the medium was aspirated and normal growth medium added.

After a 48 hour incubation at 37°C, cells were washed with 1X PBS and lysed at room temperature using 1X Passive Lysis Buffer (Promega). A total of 4 µl of the resulting cell lysate were transferred to a white polystyrene 96-well assay plate (Corning Inc., Corning, NY). Luciferase and renilla activities were determined using the Dual Luciferase Reporter 1000 Assay System (Promega) and a model Centro LB 960 luminometer (Berthold Technologies, Bad Wildbad, Germany). Each experiment was performed at least 16 times, and the ratio of luciferase to renilla activity, and the fold increase in this ratio over that observed for pLGF-E1b with no insert were calculated. The mean (bar height in figures), standard deviation (error bars in figures), and p-values (asterisks in figures) were determined using standard calculations.

### Zebrafish Reporter Gene Expression Assays

Zebrafish were raised and bred in accordance with standard conditions [38],[39]. Embryos were maintained at 28°C and staged in accordance with standard methods [38],[39]. EGFP expression constructs (see above) were injected into AB background G0 embryos (n≥200), as previously described [27],[40]. Injected embryos were evaluated for reporter expression between 24 hpf and 5 dpf. Embryos displaying consistent EGFP expression were selected and allowed to mature, facilitating germline transmission and evaluation of reporter expression. Embryos were analyzed and imaged using a Carl Zeiss Lumar V12 Stereo microscope with AxioVision version 4.5 software.

### In Situ Hybridization

Embryos selected for *in situ* hybridization were raised in embryo medium containing 0.003% phenylthiocarbamide to prevent pigmentation and fixed for *in situ* hybridization using standard protocols. Probes were labeled with digoxigenin using DIG RNA Labeling Kit (Roche Applied Science, Indianapolis, IN) and detected with the appropriate antibody. A blue precipitate was formed by incubating with BCIP and NBT (Roche Applied Science). *Green fluorescent protein (GFP)* probe was used as previously described [27].

### Transient Transgenic Mouse Analysis

Linearized plasmid DNA for microinjections was isolated using gel electrophoresis followed by electro-elution into 1X TAE buffer. DNA was further purified using Elutip-d column ion exchange chromotography (Schleicher & Schuell, Keene, NH). The resulting elutant was precipitated and resuspended into injection buffer (7.5 mM Tris-HCl, 0.15 mM EDTA, pH 8.0 at a concentration of 2 µg/ml). Microinjections into FVB-N/Tac pronuclei were performed as previously described [41].

Subsequent to injections and embryo dissection, embryos were fixed on ice in 1X PBS, 1% formaldehyde, 0.2% glutaraldehyde, and 0.02% NP40 for 2 hours, followed by 3 15-minute room-temperature washes in 1X PBS, 2 mM MgCl2, and 0.02% NP40. Embryos were then stained overnight in 1X PBS, 12 mM K-Ferricyanide, 12mM K-Ferrocyanide, 0.002% NP40, 4 mM MgCl2, and 320 µg/ml 5-bromo-4-chloro-3-indolyl-b-D-galactopyranoside in N, N-dimethyl formamide at 37°C. Embryos were then washed twice in 1X PBS and 0.2% NP40 for 30 minutes at room temperature and transferred for analysis and storage into 4% formaldehyde, 10% methanol, and 100 mM sodium phosphate.

### Genome-Wide Identification of Potential Sox10 Response Elements

To identify dimeric SoxE consensus sequences throughout the mouse genome, we wrote a Perl computer program to scan mouse genomic sequences [the February 2006 assembly (mm8) at the UCSC Genome Browser] and report any occurrence of ACAAA(N_2-10_)TKTGT, where N_2–10_ represents between 2 and 10 N's (any nucleotide) and K represents a G or T nucleotide. Subsequently, we determined the genomic context of each identified sequence (intronic, exonic, upstream of gene, downstream of gene) using the Table Browser at the UCSC Genome Browser (http://genome.ucsc.edu/cgi-bin/hgTables).

To identify genomic sequences similar to *Sox10*-MCS4 and *Sox10*-MCS7, we examined the conservation profile of these two genomic segments at the UCSC genome browser. This revealed that each genomic segment had a PhastCons conservation score of at least 400 (genome.ucsc.edu). The Table Browser at the UCSC Genome Browser was then employed to find genomic segments that: (1) harbor dimeric SoxE consensus sequences (see above); (2) have a conservation score of at least 400; and (3) reside within an intron. The gene name and RefSeq identifier for each gene was then extracted from the Table Browser. This criteria was subsequently compared to a melanocyte expressed gene list, which was generated as defined by >2-fold higher expression in melan-a as compared to 3T3 on custom printed Operon (Huntsville, Al) Mv3.0 oligonucleotide probe set representing over 16,000 genes (data not shown). The non-coding genomic segments at each of the identified loci were then assessed for enhancer activity in cultured melanocytes and Schwann cells as described above.

## Supporting Information

Table S1 Highly conserved intronic regions harboring SoxE consensus sequences.
(0.08 MB DOC)
