# Supplementary material for: Identification of Neural Crest and Glial Enhancers at the Mouse Sox10 Locus through Transgenesis in Zebrafish
Source: PLoS Genet. 2008 Sep 5;4(9):e1000174. doi: 10.1371/journal.pgen.1000174 (PMC2518861; doi:10.1371/journal.pgen.1000174)
Supplement: Table S1 — Highly conserved intronic regions harboring SoxE consensus sequences. (0.08 MB DOC) [file pgen.1000174.s001.doc]

**Supplemental Table 1.** Highly conserved intronic regions harboring SoxE consensus sequences

| Element Number | Locus Name | Coordinates at UCSC Genome Browser^1^ |
| --- | --- | --- |
| 1 | Eya1 | chr1:14170081-14170447 |
| 2 | Eya1 | chr1:14178019-14178266 |
| 3 | Pax3 | chr1:77997460-77997799 |
| 4 | Sdccag8 | chr1:178674522-178674999 |
| 5 | Sdccag8 | chr1:178718465-178718630 |
| 6 | Sdccag8 | chr1:178756413-178756667 |
| 7 | Spata17 | chr1:188842529-188842843 |
| 8 | 2610528K11Rik | chr2:33618005-33618411 |
| 9 | Dennd1a | chr2:37835571-37836036 |
| 10 | Ctnnd1 | chr2:84404012-84404139 |
| 11 | Mrg1 | chr2:115734174-115734542 |
| 12 | Ctnnbl1 | chr2:157468257-157468584 |
| 13 | Veph1 | chr3:66187869-66187985 |
| 14 | Slc26a7 | chr4:14547331-14547477 |
| 15 | F730047E07Rik | chr4:24648813-24649503 |
| 16 | Klhl32 | chr4:24799576-24799698 |
| 17 | Nfia | chr4:97418811-97419207 |
| 18 | Lcorl | chr5:46057473-46057940 |
| 19 | Ccni | chr5:94271154-94271521 |
| 20 | Cutl1 | chr5:136719226-136719658 |
| 21 | St7 | chr6:17879316-17879781 |
| 22 | Sfxn5 | chr6:85183821-85184538 |
| 23 | Fancd2 | chr6:113530389-113530659 |
| 24 | Atp6v1e1 | chr6:120783416-120783647 |
| 25 | Xylt1 | chr7:117347395-117347681 |
| 26 | Tox3 | chr8:93192308-93192803 |
| 27 | Zbtb16 | chr9:48430373-48430794 |
| 28 | Zbtb16 | chr9:48503867-48504266 |
| 29 | Rbms3 | chr9:117016226-117016388 |
| 30 | Odz1 | chrX:38984409-38984582 |
| 31 | Odz1 | chrX:39039750-39040204 |
| 32 | Odz1 | chrX:39288012-39288296 |
| 33 | Dmd | chrX:79320055-79320708 |
| 34 | Dmd | chrX:80781335-80781560 |
| 35 | Sh3kbp1 | chrX:155184526-155184652 |
| 36 | Ranbp17 | chr11:33200585-33201475 |
| 37 | Ranbp17 | chr11:33243041-33243736 |
| 38 | Odz2 | chr11:36264423-36264625 |
| 39 | Odz2 | chr11:36404372-36404901 |
| 40 | Odz2 | chr11:36427461-36427981 |
| 41 | Acaca | chr11:84153154-84153609 |
| 42 | Acaca | chr11:84166053-84166239 |
| 43 | Acaca | chr11:84184420-84184756 |
| 44 | Bcas3 | chr11:85208706-85208922 |

^1^Coordinates are from the February 2006 UCSC Genome Browser Mouse assembly
